# Editorial: Translational Advances in Alzheimer's, Parkinson's, and Other Neurodegenerative Dementias

**DOI:** 10.3389/fnagi.2022.858467

**Published:** 2022-03-17

**Authors:** Jiehui Jiang, Kuangyu Shi, Fangyu Peng, Chih-Yu Hsu, Woon-Man Kung

**Affiliations:** ^1^School of Life Science, Institute of Biomedical Engineering, Shanghai University, Shanghai, China; ^2^Department of Nuclear Medicine, Inselspital, Bern University Hospital, University of Bern, Bern, Switzerland; ^3^Department of Informatics, Technical University of Munich, Munich, Germany; ^4^Department of Radiology, The University of Texas Southwestern Medical Center, Dallas, TX, United States; ^5^Fujian Provincial Key Laboratory of Big Data Mining and Applications, School of Computer Science and Mathematics, Fujian University of Technology, Fuzhou, China; ^6^Division of Neurosurgery, Department of Surgery, Taipei Tzu Chi Hospital, Buddhist Tzu Chi Medical Foundation, New Taipei City, Taiwan

**Keywords:** Alzheimer's disease, Parkinson's disease, dementia, neurodegenerative disorders, big data mining, imaging methods, bioinformatic applications

The increase in number of people suffering from neurodegenerative dementia (ND), such as Alzheimer's disease (AD) and Parkinson's disease (PD), has placed a huge burden on society. Precise detection of ND in an early phase is still challenging in daily clinical practice (Tisher and Salardini, 2019). There is a continuous escalation in effort from more researchers to investigate molecular mechanism of ND pathophysiology and develop new biomarkers for diagnosis and treatment of ND, as reflected by significant numbers of articles contained in this Research Topic.

In addition to metabolic changes such as altered glucose metabolism, alterations of amyloid-beta and tau protein are biomarkers associated with pathology of AD, whereas alpha-synuclein is proven to be related to PD and other NDs (Congdon and Sigurdsson, 2018; Rocha et al., 2018; Butterfield and Halliwell, 2019; Pinheiro and Faustino, 2019). Molecular imaging techniques using radiotracers like 2-deoxy-2-[^18^F]fluoroglucose (^18^F-FDG), 4-[(E)-2-(6-{2-[2-(2-(^18^F)fluoroethoxy)ethoxy]ethoxy}pyridin-3-yl)ethen-1-yl]-N-methylaniline (^18^F-AV45), and 7-(6-(^18^F)fluoropyridin-3-yl)-5H-pyrido[4,3-b]indole (^18^F-AV1451) have been widely used in research and diagnostic imaging of ND. Development of bioinformatics analysis and artificial intelligence methods has significantly facilitated advances in research of ND. Nowadays, researchers are facing difficulties in choosing the best method in order to conduct research in a comprehensible fashion with estimates of accuracy and reproducibility.

Taking this into consideration, the present Research Topic on “*Translational Advances in Alzheimer's, Parkinson's, and Other Neurodegenerative Dementias”* by Frontiers in Aging Neuroscience made a contribution with updates and different perspectives on this important theme to contain over 69 papers. These updates focus on exploring reliable biomarkers and prediction indexes for the progression of ND from multidisciplinary perspectives. In addition, researches in development of novel therapies were also included.

Based on univariate and multivariate analyses, Wei et al. found that the apolipoprotein E (APOE) e4 allele might affect the relationship between serum lipid levels and cognitive impairment. In addition, Hu, Li, Zhao, et al. provided evidence that the combination of telmisartan and rosuvastatin might be an effective prevention and/or treatment strategy for cognitive impairment and dementia, especially in hypertensive patients with the APOE e4 allele. Kuang et al. demonstrated that the e4 genotype leads to distinct default mode network (DMN) functional alterations in early phases of AD using persistent homology approach. Lin investigated whether CDGSH iron-sulfur domain 2 (CISD_2_) gene attenuation had an influence on anti-inflammatory effects and M1-M2 polarization in microglia. This study promised a potential therapeutic target for ND.

For clinical diagnosis of AD, Jiao et al. focused on plasma biomarkers which are less expensive and invasive than those necessitating a spinal fluid sample. The results of their study showed that the multifactor model of plasma amyloid-beta 42 and total-tau in combination with Montreal Cognitive Assessment (MoCA) could be a viable model separate health and AD subjects in clinical practice. Gao et al. characterized the relationship between plasma amyloid-beta levels and cognitive decline in 1,240 cognitively normal participants. The relationship between plasma amyloid-beta 40 and cognitive decline was an inverted-U shape in a cognitively normal population. None of relationship between plasma amyloid-beta 42, amyloid-beta 42/40, and cognitive decline was found during a 2-year follow-up. Lin et al. enrolled cognitively normal amyloid-beta-positive participants from 2 cohort studies, all types of resilience to cerebrospinal fluid (CSF) amyloid-beta could predict longitudinal cognitive decline.

Identification of novel molecular biomarkers for diagnosis and treatment of AD is urgently demanded. Based on bioinformatics analysis, Yu et al. identified 16 hub genes correlated to the neuropathological stage and 35 potential biomarkers for the diagnosis of AD. Yuen et al. demonstrated a systematic workflow for evidence synthesis of transcriptomic studies using both meta-analysis and bioinformatics methods to identify potential pathogenic factors. The results showed that reduced amyloid-beta clearance in AD pathogenesis was associated with genes encoding Fyn and EGFR, which were key receptors in amyloid-beta downstream signaling. Robin et al. comprehensively profiled phenotypic features over time in one commercially-available induced Pluripotent Stem Cell (iPSC)-derived human neuron cell line. This study provided a tool to investigate neurodegenerative and other central nervous system (CNS) diseases. Deng et al. applied multivariate model to estimate the association between leukocyte telomere length (LTL) and cognitive performance. Their results suggested that LTL might be a biomarker of cognitive aging.

Kong et al. explored the links between diabetes and AD by studying the advanced glycation end products (AGEs) and the receptors for AGEs (RAGE). The results of their study suggested that patients with diabetes were at a higher risk of developing AD. They further reviewed the interaction between RAGE and amyloid-beta as well as tau, which highlighted the potential of RAGE to be used as an effective target for AD diagnosis and treatment. Ho et al. explored type 2 diabetes mellitus (T2DM) pathogenesis in the amyloidogenic evolvability. A better understanding of the role of T2DM in amyloidogenic evolvability might reveal new targets for therapeutic intervention in AD patients who are comorbid with T2DM.

Using magnetic resonance (MR) imaging, Li et al. found that T2DM could give rise to the white matter atrophy of several brain regions, including left posterior cingulate, precuneus, insula, and right rostral middle frontal gyrus. In addition, they investigated the white matter structural network disruption in T2DM patients with MCI. Chen et al. developed MR glucose chemical exchange saturation transfer (glucoCEST) imaging in a rat model of AD. The findings from their study showed that this method could explore the occurrence and progress of diabetes-related AD or dementia.

Based on live-cell imaging combined with behavioral tests, Feng et al. explored the role and underlying mechanism of calcium-sensing receptor (CaSR) in cognitive deficits in AD mice. Their study might provide novel insights on the potential of CaSR as a therapeutic target for AD. Wu et al. examined whether steroid receptor coactivator 1 (SRC-1) is involved in pathogenesis of AD. Tyagi et al. reviewed the role of cyclooxygenases (COX) and mammalian/mechanistic target of rapamycin (mTOR) and potential therapeutic approaches targeting COX-2 and mTOR in AD and cancer.

Bjorkli et al. reviewed the preclinical and clinical investigations of commonly used biomarkers in animal models of AD and AD patients respectively. They also provided recommendations for standardization of procedures in sample collection to enhance the translational validity of preclinical study using AD animal models.

TDP-43 is a protein related to amyotrophic lateral sclerosis (ALS) and many cases of tau-negative frontotemporal lobar degeneration. Zhang, Chen, et al. reviewed the researches about TDP-43 and its relationship with limbic-predominant age-related TDP-43 encephalopathy (LATE).

In an effort to develop neurodegenerative disease model at cellular level, Zhang, Xie, et al. reported prospects of 2 kinds of reprogramming technologies for neurodegenerative diseases: (1) convert adult somatic cells to iPSCs and (2) directly reprogramming adult somatic cells to induced Neurons (iN).

Jang et al. provided updates on current progress in stem cell-derived dopaminergic neuron transplantation as a therapeutic alternative for PD. Yang, Zhang, et al. aimed to uncover the metabolic pathways across anatomical regions in the brain of PD and levodopa-induced dyskinesia (LID). Based on principal component analysis (PCA) and multivariate general linear model, the midbrain and right cortex were identified as the primary regions of metabolic abnormalities in PD and LID rats. In addition, PD and LID rats exhibited lower levels of synaptophysin (SYP). All results provided key insights for developing targeted therapies in PD. Harsanyiova et al. discussed the relationship between gastrointestinal tract and the pathology or treatment of PD symptoms.

Bao et al. surveyed the various positron emission tomography (PET) radiotracers available for AD imaging and discussed their clinical applications especially in terms of early detection and cognitive relevance. Based on [^18^F]-APN-1607 PET tracer, Lu et al. detected tau deposition in AD and reported that individual tauopathy is correlated with impaired cerebral glucose metabolism and cognitive function. Using combined PET and MR imaging, Kim et al. investigated the effect of conductive hearing loss in an AD mouse model. The findings from their study indicated that even partial hearing loss could aggravate memory impairment in AD.

^11^C-PE2I is a PET radiotracer targeting neuronal dopamine transporters (DaT). Ivanidze et al. investigated neurovascular unit (NVU) integrity by using arterial spin labeling (ASL) MR imaging and correlated the findings of NVU integrity with striatal DaT density from ^11^C-PE2I PET imaging. This exploratory research could serve as a foundation for further development of combined NVU and striatal DaT density as early disease biomarkers and potential new therapeutic targets. Based on ^11^C-CFT and ^18^F-FDG PET imaging data, Fei et al. studied the relevancy between UPDRS motor scores and PDQ39 mobility sub-scores.

Based on partial volume-coil ^31^P MR spectroscopy of parieto-occipital lobes with 7-Tesla MR imaging, Das et al. accurately quantified high-energy phosphate and membrane phospholipid metabolites in amnestic MCI (aMCI). Furthermore, they have also found that brain energy metabolism and membrane phospholipid indexes were related to cognitive performance in domains of executive function (EF), memory, attention, and visuospatial skills using aMCI.

Liu, Jiang, et al. explored functional and structural properties of abnormal brain networks associated with PD. The authors showed that both the expressions of metabolic and structural patterns in PD patients were significantly higher than healthy controls, and verified their results in connectome analysis, which provided new information for elucidating the neuropathological mechanisms of PD. Shu et al. developed an integrative nomogram based on white matter radiomics biomarkers and nonmotor symptoms for the identification of early-stage PD.

Tsai et al. explored the skull score (SS) distribution of tremor patients, and correlated the SS with image feature from customized skull density ratio (cSDR). This study provided useful information for clinical study of MR-guided focused ultrasound thalamotomy. Chiu, Tzeng, et al. found that the patients with tremor and vascular cognitive impairment (VCI) had high possibility of mixed pathology of PD and Lewy body disease (LBD).

Ye et al. investigated the volumetric changes in thalamus and hypothalamus in ALS. The results from their study revealed no significant difference of the volume in thalamus and hypothalamus between ALS patients of normal frontotemporal function and healthy controls.

Yu et al. identified the brain function activity differences between MCI patients with depression and MCI patients without depression using resting state MR imaging measurements. This study provided useful information for a better understanding of the relationship between depressive symptoms and memory deficits. Xing et al. explored the alterations in intra- and inter-network functional connectivity of multiple networks in presbycusis patients, suggesting that functional network connectivity can be used to predict potential cognitive impairment in their early stage.

Wan et al. compared the changes in subcortical nuclei in older adults with cognitive frailty (CF) and studied their relationship with cognitive decline and physical frailty. Their results showed significant volume reductions in five subcortical nuclei, including the bilateral thalami, left caudate, right pallidum, and accumbens area in older adults with CF. Lee et al. used surface-based analysis to evaluate subcortical structural characteristics and its relationship with early onset Alzheimer's disease (EOAD) and late-onset Alzheimer's disease (LOAD). The results from their study demonstrated that EOAD and LOAD might have different courses of pathomechanism.

Qiao et al. examined the neural substrates and mechanisms that generate memory deficits, seizures, and neuropsychiatric abnormalities in encephalitis with leucine-rich, glioma-inactivated 1 (LGI_1_) antibodies. The results showed that neural disorder and behavioral deficits of anti-LGI_1_ encephalitis might be associated with extensive changes in brain connectivity and microstructure.

Bruchhage et al. performed machine learning to assess the contribution of volume fraction myelin and gray matter volume. They proposed a refined model of cerebellar contribution to early AD development. The results from their study showed higher anterior cerebellar contribution to MCI and higher posterior cerebellar contribution to mild/moderate stages of AD for each tissue property. Ma et al. proposed a generative adversarial network (GAN) framework to distinguish brain images of human subjects of normal brain aging from those of human subjects with AD and frontotemporal dementia (FTD). The results from their study showed an accuracy of 88.28% in distinguishing human subjects of normal brain aging from those of human subjects with AD and FTD based on GAN framework derived from brain image.

Hu, Li, Zhang, et al. found that periodontitis was associated with learning and memory impairment, probably induced by neuroinflammation via activating the toll-like receptor 4/Nuclear factor kappa B signaling pathway. Ding et al. aimed to evaluate the value of odors in olfactory identification (OI) test and other known risk factors for predicting incident dementia. The results from their study suggested that peppermint smell capability might be one of the useful indicators for predicting dementia. Nie et al. assessed the differences of eye movement parameters between healthy elderly individuals and patients with MCI. They found that cognitive deficits and eye movement indexes were correlated, which could be further explored as early markers for MCI.

The paper by Yang, Huang, et al. proposed a neuroimaging approach to identify MCI using a deep learning method and functional near-infrared spectroscopy (fNIRS). Their results indicated that fNIRS imaging approach based on temporal feature maps as a promising diagnostic method for early detection of MCI and clinicians might use it as a tool for evaluation of MCI.

Tseng et al. used multivariate time-series electrocardiogram (ECG) analysis to diagnose cardiovascular diseases. They investigated various ECG features and found some associations between features of ECG and medical records (e.g. smokers, obesity, and hypertension).

Chiu, Hung, et al. studied the relationship between freezing of speech (FOS) and dementia with Lewy bodies (DLB). They designed a freezing of speech single questionnaire (FOSSQ) and compared the association factors of FOS in non-demented participants, patients with AD, vascular dementia (VaD), and DLB. The results of their study supported validity of the FOSSQ for discriminating DLB from individuals with non-demented or other forms of dementia. Similarly, Wang, Hung, et al. designed a novel questionnaire for visuospatial dysfunction (VSD) in DLB.

To distinguish stable MCI (MCIs) from converting MCI (MCIc), Gupta et al. proposed different neuroimaging modalities combined with APOE genotype to form a multimodal system for discrimination of AD to increase the classification accuracy.

In order to determine whether a MCI patient is at high risk of progressing to AD, Lin et al. developed an extreme learning machine (ELM)-based grading method to predict MCI-to-AD conversion. The method was validated by Alzheimer's Disease Neuroimaging Initiative (ADNI) cohort, with an accuracy of 84.7% in prediction of MCI progression to AD within 3 years.

Wang, Wei, et al. conducted a systematic review and meta-analysis to assess the available preclinical evidence and possible mechanisms of baicalein for animal models of PD. Li et al. investigated the efficacy and safety of 3 Erinacine A-enriched Hericium erinaceus mycelia (EAHE) capsules for the treatment of patients with mild AD. The study lasted for 49 weeks and the results showed that EAHE was well-tolerated without significant side effects. Flavonoid containing natural products, Myricetin (MYR) and Dihydromyricetin (DMY) are abundant in fruits and vegetables. Liu, Guo, et al. reviewed the benefits of MYR and DMY in AD patients at molecular level, including effects on amyloid-beta protein imbalance, neuroinflammation, dyshomeostasis of metal ions, autophagy disorder, and oxidative stress.

The early intervention for MCI could decrease the rate of conversion from MCI to AD. Lai et al. compared and ranked 9 treatment methods for MCI in AD based on meta-analysis. They pointed out that music therapy might be the best treatment for MCI followed by acupuncture, among the nine treatment methods included for their meta-analysis. Similar to those non-pharmacological interventions, Pei et al. proposed a neurofeedback training based on mismatch negativity to regulate sensory ability and memory.

Stuckenschneider et al. investigated the effects of a 12-month structured exercise program on the progression of 183 amnestic MCI patients. No significant improvement of cognitive performance was found based on the results from their study. In another study based on event-related measurement of auditory memory, Laptinskaya et al. found no improvements of cognitive performance in a 10-week unimodal cognitive or physical training and an active lifestyle for older adults at risk for dementia. In the narrative review by Meng et al., patients with AD who presented with long-term exercise interventions appeared to have improved blood flow, increased hippocampal volume, and improved neurogenesis. These results indicated that exercise intervention might be an important moderator to prevent long term disease progression.

Kumar et al. reviewed and discussed nano-enabled drug delivery systems and their current and potential applications for the treatment of various NDs, including AD and PD, in studies to overcome the limits of blood-brain barrier (BBB). Huang et al. developed a medical red light treatment (RLT) device to treat older adults with mild to moderate AD. They planned a study protocol to verify the safety and efficacy for a 24 weeks period. On the other hand, Zhu et al. found that there was more efficacy via tele-health interventions in lowering depression for careers of dementia patients based on meta-analysis.

Deep brain stimulation (DBS) is widely used in the field of mental and neurological diseases. Luo et al. reviewed the therapeutic effect of DBS in AD, and analyzed its stimulation parameters and potential mechanism of action. Tan et al. used a convolutional neural network (CNN) to classify handwritten digits and letters and applied dropout at different stages to simulate DBS effects on engrams. The results of their study showed that dropout of engram nodes might be a possible mechanism by which neuromodulation techniques could disrupt or enhance memory. Hwang et al. pointed out the applications of phase amplitude coupling-based phase-dependent DBS technique in PD, which aimed to deliver timed stimulation pulses to a specific phase precisely to modulate pathological network activities and behavior in real time.

Tang et al. investigated the effect of electroacupuncture (EA) on cognitive impairment and the role of c-Jun N-terminal kinase (JNK) signaling pathway in AD model mice. The results of their study showed that EA could reverse cognitive deficits and substantially lower the burden of amyloid precursor protein. In another study, Hongna et al. demonstrated the improvements of locomotor function by promoting autophagy flux and inhibiting necroptosis in rats with spinal cord injury treated with Jia-Ji electro-acupuncture.

In summary, large number of articles collected in this Research Topic reflected recent advances in mechanistic study of pathophysiology of AD and PD, development of biomarkers and molecular imaging techniques for diagnosis and treatment of AD and PD. We hope that publications of this Research Topic will not only report recent advances in ND research, but also facilitate translation of new discovery to development of new diagnostic tests and therapeutic agents for early diagnosis and treatment of ND, including AD and PD.

## Author Contributions

JJ and KS wrote the draft. FP copyedited for the language. C-YH and W-MK reviewed and revised the manuscript. All authors listed have made a substantial, direct, and intellectual contribution to the work and approved it for publication.

## Conflict of Interest

The authors declare that the research was conducted in the absence of any commercial or financial relationships that could be construed as a potential conflict of interest.

## Publisher's Note

All claims expressed in this article are solely those of the authors and do not necessarily represent those of their affiliated organizations, or those of the publisher, the editors and the reviewers. Any product that may be evaluated in this article, or claim that may be made by its manufacturer, is not guaranteed or endorsed by the publisher.

## References

[B1] Butterfield D. A. Halliwell B. (2019). Oxidative stress, dysfunctional glucose metabolism and Alzheimer disease. Nat Rev Neurosci. 20, 148–160. 10.1038/s41583-019-0132-6 30737462 PMC9382875

[B2] Congdon E. E. Sigurdsson E. M. (2018). Tau-targeting therapies for Alzheimer disease. Nat Rev Neurol. 14, 399–415. 10.1038/s41582-018-0013-z 29895964 PMC6463489

[B3] Pinheiro L. Faustino C. (2019). Therapeutic strategies targeting amyloid-beta in Alzheimer's disease. Curr Alzheimer Res. 16, 418–452. 10.2174/1567205016666190321163438 30907320

[B4] Rocha E. M. De Miranda B. Sanders L. H. (2018). Alpha-synuclein: pathology, mitochondrial dysfunction and neuroinflammation in Parkinson's disease. Neurobiol Dis. 109, 249–257. 10.1016/j.nbd.2017.04.004 28400134

[B5] Tisher A. Salardini A. (2019). A comprehensive update on treatment of dementia. Semin Neurol. 39, 167–178. 10.1055/s-0039-1683408 30925610

